# Women's health and rights in conflict: the impact of renewed violence in Lebanon

**DOI:** 10.1080/26410397.2025.2506263

**Published:** 2025-05-16

**Authors:** Faysal El Kak

**Affiliations:** Clinical Associate of Obstetrics, Gynecology, Sexual Health, and Senior Lecturer of Public Health, Director of Women Integrated Sexual Health Program, American University of Beirut, Beirut, Lebanon.

**Keywords:** conflict, displacement, sexual reproductive health and rights, mental health, gender-based violence

## Background and context

The renewed violence in Lebanon since 2024, exacerbated by ongoing airstrikes linked to the regional conflict in Gaza since 2023, has caused widespread disruption, displacement, and devastation. These events mark an escalation of hostilities that began in 2023, following decades of intermittent instability. In Lebanon, the renewed violence has not only led to infrastructure collapse but has also displaced over 1.2 million individuals within a few weeks, disproportionately affecting women and children.^1^ This situation has compounded existing vulnerabilities as Lebanon grapples with an economic crisis and strained healthcare infrastructure. Understanding the everyday realities of displaced populations and the specific challenges women face is critical to addressing health and rights impacts. Amongst the massive number of displaced personnel, there were more than 250,000 internally displaced persons (IDPs) staying in crowded shelters (1200 shelters), mostly schools, religious places, deserted houses, and NGO spaces in cities and towns. Many shelters lack proper construction and maintenance, offering little protection from the elements. Basic amenities, including access to clean drinking water, functioning toilets, waste disposal facilities, and privacy, are also lacking. Reports indicate that displaced families often share small spaces with multiple strangers, increasing risks to personal safety, especially for women and girls, placing disproportionate stress on reproductive and sexual health and increasing the risk for gender-based violence (GBV) and mental health issues.^2^

It is important to mention that most shelters receive allotted supplies of water, food ration (sometimes cooked food), and basic hygiene kits. It was interesting to note that women in the shelters reorganised themselves around dynamics and practices they used to do in their villages before displacement, and that included sharing household work, cooking, and childcare (including activities and art classes) through division of labour and supporting each other. Women reported feeling safe in the shelters as they don't have to travel or walk long distances to get food or water.

The health impact of the conflict on the IDPs is deleterious, especially on women and children who represent more than 50% of the total. Among the IDPs, there were an estimated 11,600 pregnant women, with about 310 expected to give birth.^3,4^ These pregnant women faced significant barriers in accessing antenatal care, safe delivery services, and postpartum care, including counselling support. There are significant concerns for pregnant women, who need special care due to interruptions and even a lack of antenatal check-ups and supervision. This may increase the risk of maternal complications, including preeclampsia, gestational diabetes, preterm birth, and infections, along with disruptions in the supply of iron and multivitamin supplements. Another concerning issue is access to safe delivery sites with skilled attendance. Although Lebanon enjoys a widely spread network of hospitals, maternity centres, and primary health care centres, access might be compromised due to continuous airstrikes and the closure of dozens of clinics,^5^ leading to higher rates of maternal and neonatal morbidity and mortality. In the postpartum period, women and newborns are at risk of infection with severe morbidity and mortality due to lack of access to clean water, and essential medications, and difficulty breastfeeding in displaced settings.^6^

Another important reproductive health concern impacted by war is menstrual health. Women and girls faced challenges accessing sanitary products. Women in IDP shelters complained of overcrowding and lack of privacy and safe spaces, preventing them from taking care of their menstrual health and personal hygiene. Limited water supply further compromises their dignity and well-being in handling personal hygiene.

Contraception and family planning are another major concern for an estimated 60,000 Lebanese displaced women of reproductive age, with many of them having compromised or poor access to primary healthcare and being at risk of unintended pregnancy.^7^ Although the supply chain remains intact, according to the Ministry of Public Health,^8^ and short and long-acting methods, including emergency contraception, are available, physical inaccessibility and the stigma around contraception within the shelter context are both considered significant cultural and social barriers. Despite the distribution of food and some basic cleansing agents to IDPs in shelters, no condoms were provided, and renewal of contraceptives was difficult.

The enormous influx of the 1.2 million displaced persons from various areas of Lebanon affected by airstrikes is thought to increase the risk of GBV for displaced women and girls due to the increase in overcrowded common shelters and a lack of access to essential resources which is related to increased stress and tension, lack of safety and privacy, and weakened social and health structures, as well as power imbalances, economic dependency, and lack of access to support services. GBV has risen sharply in the wake of Lebanon's war, with more than half a million displaced women and girls being particularly vulnerable.^9^ Reports of sexual violence, domestic abuse, and human trafficking have escalated as women face insecurity in both public and private spaces. Overcrowded shelters, where families live in close quarters with strangers, expose women to heightened risks of exploitation and abuse.^10^

There are currently 12,000 displaced families that are female-headed, and these women must provide for their children and other family members, which puts them at higher risk of harassment and abuse.^9^ These circumstances further strip women and girls of their rights and expose them to long-term physical and emotional trauma.

Conflict severely impacts mental health, especially among women facing trauma from displacement, GBV, and family separation. In Lebanon, the current instability has significantly amplified these mental health challenges, particularly among internally displaced individuals and refugee populations. Women in Lebanon are exposed to multiple psychological stressors, from economic hardship to violence. Pregnant women and new mothers, in particular, struggle with depression, anxiety, panic attacks, and postpartum depression.^11^ Efforts by organisations, including the national programme on mental health at the Ministry of Public Health, have been mobilised to support women and displaced persons to address these urgent needs, particularly in conflict-prone regions and shelters.^12–14^

Several interventions are in place to support internally displaced persons (IDPs), aiming to provide food, water, hygiene kits, medical visits, mental health and psychosocial support, and free-of-charge clinical services. In particular, the MoPH initiated rape–survivor support in shelters, an initiative taken for the first time since past conflicts. The Women Integrated Sexual Health (WISH) Program at the American University of Beirut, in collaboration with OXFAM-Novib, conducted several interventions around health promotion. These included SRHR training of community-identified champions to support the women and children in shelters, health promotion sessions, and training of healthcare providers on SRHR in emergencies.^14^

## Conclusion and recommendations

The renewed violence in Lebanon has compounded existing economic, financial, and healthcare challenges, creating a protracted dire situation for displaced women and girls. The intersection of conflict, displacement, and gender-specific vulnerabilities has resulted in a surge of reproductive health and gender-based violence risks, and mental health crises. Existing support structures – though vital – remain insufficient in reach and capacity to mitigate and eliminate those risks and issues.

To move from crisis response to sustainable support, a more integrated, proactive, and gender-sensitive humanitarian approach is urgently needed. The following actionable recommendations can be helpful:

1. **Deploy mobile and community-based health services**

Expand mobile clinics and deploy trained community health workers to deliver antenatal, postpartum, and contraceptive care directly within shelters and displacement zones. These must include culturally sensitive education and outreach efforts.

2. **Ensure safe delivery and emergency obstetric services**

Establish and strengthen temporary maternal health units or partner with unaffected facilities to create designated safe zones for childbirth and emergency care, reducing risks associated with home or shelter-based deliveries.

3. **Distribute dignity kits with menstrual and hygiene supplies**

Regularly sustain provision of well-stocked dignity kits that include sanitary pads, underwear, soap, and culturally appropriate instructions on menstrual hygiene management and safe spaces, especially for adolescent girls.

4. **Strengthen GBV response and survivor protection**

Scale up GBV referral networks, deploy trained female protection officers to shelters, and ensure the availability of legal, psychosocial, and medical services – particularly in under-resourced areas.

5. **Integrate mental health into all humanitarian services**

Strengthen the existing National Health Program on Mental Health to embed mental health and psychosocial support into all levels of care, with a focus on trauma-informed services for women and adolescent girls experiencing violence or distress.

6. **Centre women's voices in humanitarian planning**

Actively involve displaced women in designing, implementing, and monitoring aid interventions to ensure that their priorities and safety concerns shape the response.

A gender-responsive approach, where programming includes specific action aiming to reduce gender inequalities within communities. must not be viewed as secondary but as essential to any effective humanitarian strategy. Safeguarding the health and rights of women and girls in Lebanon is a matter of urgency – and dignity. The current crisis offers an opportunity to build a more equitable, inclusive system of care that recognises women not only as recipients of aid but as central agents of resilience and recovery.

## Conclusion

The ongoing violence in Lebanon presents significant challenges to women's health and rights, particularly among displaced populations. Pregnant women, survivors of gender-based violence, and those with chronic health conditions face particularly acute challenges. Lebanon's healthcare system, stretched thin by conflict and economic hardship, currently relies heavily on the dedication of the international community and local organisations to sustain a humanitarian response and offer essential services tailored to women's needs. The Ministry of Public Health, through the primary health care system, should adopt a more inclusive and resilient healthcare approach that incorporates maternal health, contraception access, GBV protections, and mental health support, not only to address immediate needs but also to build a foundation for long-term healthcare stability. This approach is critical for upholding women's dignity and safeguarding their well-being during one of the country's most challenging times in modern history.
